# Harnessing mitochondrial biogenesis to combat acute kidney injury: Current insights and futuredirections

**DOI:** 10.1016/j.gendis.2025.101645

**Published:** 2025-04-15

**Authors:** Yajie Hao, Fahui Chen, Xiya Ren, Xiu Huang, Xiaoshuang Zhou

**Affiliations:** a The Fifth Clinical Medical College of Shanxi Medical University, Taiyuan, Shanxi 030000, China; b The Third Clinical College, Shanxi University of Chinese Medicine, Jinzhong, Shanxi 030600, China; c Department of Nephrology, Shanxi Provincial People's Hospital, The Fifth Clinical Medical College of Shanxi Medical University, Shanxi Kidney Disease Institute, Taiyuan, Shanxi 030000, China

**Keywords:** Acute kidney injury, Mitochondrial biogenesis, Small compounds, Therapeutic interventions, Translational medicine

## Abstract

Mitochondrial biogenesis (MB) is involved in the regulation of cellular energy metabolism, stress response, and survival. This review examines therapeutic approaches to acute kidney injury (AKI) that target MB, emphasizing clinical research findings and translational strategies in this field. AKI is a severe condition with high mortality and often leads to chronic kidney disease. AKI suppresses MB, resulting in mitochondrial dysfunction, oxidative stress, and further renal damage. Furthermore, studies have shown that ischemia-reperfusion-, sepsis-, and drug-induced AKI inhibit MB and subsequent kidney injury. Studies have shown that targeting MB through genetic and pharmacological interventions can alleviate AKI by restoring mitochondrial function and improving renal outcomes. Small molecule compounds, such as pyrroloquinoline quinone, ZLN005, and resveratrol, can enhance MB, offering potential therapeutic benefits. Nonetheless, further studies are needed to ensure efficacy across different models and mitigate related side effects. Future research should focus on optimizing drug design, understanding MB regulation, and conducting clinical trials to establish effective treatments for AKI.

## Introduction

Acute kidney injury (AKI) is characterized by an abrupt decline in kidney function and is associated with a high risk of mortality. Studies have suggested that AKI is related to significant long-term adverse outcomes, including increased mortality rates and the development of chronic kidney disease.^1^ The mortality rate associated with AKI is more than fourfold higher than the rate in patients without AKI.^2^ Furthermore, AKI that requires renal replacement therapy has a mortality rate exceeding 50% in intensive care settings, higher than the mortality rates of other serious conditions, such as acute lung injury and myocardial infarction.^3^ The high mortality rates among AKI survivors, even those who do not require chronic dialysis post-discharge, indicate an ongoing risk of death, with AKI survivors facing an elevated likelihood of entering chronic dialysis.^4^ The mortality rate of AKI in critically ill children with AKI is also high, highlighting the importance of understanding and addressing AKI across all age groups.^5^ Therefore, effective preventative measures, early detection methods, and treatment strategies are needed for AKI to reduce its prevalence, associated mortality, long-term dialysis, and related healthcare costs.

Mitochondria, also known as the powerhouses of the cell, play a critical role in regulating cellular functions and have become key targets in disease-related research. Mitochondrial dysfunction promotes the development of various diseases, including cardiovascular diseases, neurological disorders, metabolic conditions, and cancer.^6^, ^7^, ^8^, ^9^ Kidney, as a high-energy-consuming organ, particularly renal tubular epithelial cells, is closely associated with mitochondrial dysfunction, leading to the development of AKI.^10^^,^^11^ Therefore, mitochondrial-targeted therapies have been developed through the regulation of mitochondrial function and maintenance of its stability under stress conditions. MB refers to the development of new mitochondria from pre-existing ones. MB involves the coordinated expression of mitochondrial and nuclear genes, the synthesis of mitochondrial DNA (mtDNA) and proteins, and the import of these proteins into the mitochondria. Various key factors, such as peroxisome proliferator-activated receptor gamma coactivator 1 alpha (PGC-1α), nuclear respiratory factor-1 (NRF1), nuclear respiratory factor-2 (NRF2),^12^^,^^13^ and mitochondrial transcription factor A (TFAM), activate and regulate MB through various signaling pathways to maintain normal cellular functions.^14^^,^^15^ Cells can increase MB to regulate ATP production, oxidative stress, and cellular homeostasis under stress conditions, thereby enhancing their resistance to external stimuli and mitigating kidney damage, promoting recovery.^16^, ^17^, ^18^ However, excessive activation of MB can lead to the misfolding of mitochondrial proteins, resulting in adverse effects. Therefore, the proper regulation of MB to maintain cellular stability under stress is crucial for targeted MB therapies. Besides, research has suggested that MB targeting can alleviate AKI.^19^^,^^20^ Also, several studies have shown that AKI is associated with MB, highlighting the potential of MB as a novel therapeutic target for AKI. This review analyzes and describes the general processes and regulatory mechanisms of MB and its association with AKI and discusses compounds targeting MB, highlighting its potential to treat AKI. Moreover, the clinical studies investigating the mitochondria-targeted therapies in the prevention of AKI damage and strategies for advancing translational medicine are described, aiming to bridge the gap between experimental research and clinical application.

## MB and its regulation

MB is a process by which cells increase the number and quality of mitochondria to adapt to changing energy demands. MB plays a critical role in cellular energy metabolism, adaptation to environmental changes, and response to various physiological and pathological states.^21^^,^^22^ MB is a complex and highly regulated process involving dual control by nuclear and mitochondrial genomes. MB involves the import of over 1000 mitochondrial precursor proteins from the cytoplasm, with more than 90% of these nuclear-encoded proteins entering mitochondria via the translocase of the outer membrane (TOM) complex.^23^ Mitochondrial precursor proteins are imported through five main transport pathways, with about 60% entering via N-terminal presequences.^24^ These proteins are synthesized in the cytoplasm and contain N-terminal presequences as targeting signals. The presequences (length: 15 to 50 amino acids) can form an amphipathic α-helix with a positively charged and hydrophobic surface.^25^ Precursor proteins are initially recognized by the receptor protein Tom20 of the TOM complex, which specifically identifies the hydrophobic surface of the presequence. The presequence is then transferred to the central receptor Tom22, which recognizes the positively charged surface. The presequence then passes through a channel formed by Tom40 into the mitochondrial intermembrane space. The precursor protein is recognized by the translocase of the inner membrane (TIM) 23 complex once in the intermembrane space. Tim50 acts as a presequence receptor, binding the precursor proteins from the Tom40-Tom22 channel. Tim23 forms a channel in the inner membrane, while Tim21 regulates this process, allowing the precursor protein to enter the mitochondrial matrix. The mitochondrial processing peptidase cleaves off the presequence.^26^ The cleaved protein is then properly folded into its mature, active form with the assistance of the Hsp60-Hsp10 chaperone complex.^27^ Additionally, mtDNA encodes some key respiratory chain complex proteins. mtDNA, located in the mitochondrial matrix, encodes 13 subunits of the respiratory chain complexes, 22 mitochondrial tRNAs, and 2 rRNAs. Transcription and translation of mtDNA occur independently within the matrix, and these gene products are directly integrated into the respiratory chain complexes.^28^ However, excessive MB activation can lead to the import of an excessive number of proteins, surpassing the folding and quality control capabilities of mitochondria, thus resulting in protein misfolding and aggregation. Misfolded proteins can form toxic aggregates, impair mitochondrial function, and induce cytotoxicity. Therefore, maintaining the balance of MB is crucial for cellular health and normal function.

Multiple signaling pathways, including adenosine monophosphate-activated protein kinase (AMPK), sirtuin 1 (SIRT1), and PGC-1α, have been shown to regulate MB by modulating the expression of associated genes in response to changes in cellular metabolic states. Cells can effectively increase the number and functionality of mitochondria to meet various physiological demands through these complex regulatory mechanisms.^15^ PGC-1α is currently considered the primary regulator of MB. PGC-1α can enhance the transcriptional activity of multiple transcription factors and interact with NRF1/2. NRF1 is a common activator of many nuclear genes encoding mitochondrial respiratory functions, including RC4, COX-VIc-2, and MRP RNA genes. NRF1 influences a single respiratory complex as well as the expression of multiple respiratory chain complexes and genes related to mitochondrial function, serving as a positive regulatory gene for MB.^29^ NRF2 also plays a crucial role in the promoter regions of respiratory chain-related genes, where it binds to these promoter regions and promotes gene transcription. For instance, NRF2 promotes the transcription of the COXVb gene by binding to and activating E26 transformation-specific (ETS) domain-binding sites within its promoter. The ETS domain is a highly conserved DNA-binding motif widely observed in the ETS family of transcription factors. By specifically recognizing DNA sequences containing the GGAA core motif, known as ETS binding sites, this domain plays a crucial role in the regulation of the transcriptional activity of target genes.^30^ Additionally, NRF2 directly or indirectly interacts with many genes related to mitochondrial respiratory functions. Genes encoding COX subunits and subunits of complexes I, II, and V, participating in the regulation of their expression, have NRF2 binding sites.^31^ Furthermore, NRF1/2 promotes the expression of genes necessary for mtDNA replication and transcription, including TFAM. TFAM directly binds to mtDNA, causing DNA bending and activating transcription from specific promoters. TFAM plays a vital role in the packaging, stabilization, and replication of mtDNA.^32^ Additionally, PGC-1α promotes the expression of genes related to mitochondrial oxidative phosphorylation by activating the orphan nuclear hormone receptor (ERRα).^33^ The nuclear factor erythroid 2-related factor 2 (NFE2L2) is a critical cellular redox regulator that, as a transcription factor, binds to antioxidant response element (ARE) in the promoters of antioxidant genes, enhancing their transcription to exert antioxidant effects.^34^ NFE2L2 also promotes MB, and findings from experimental studies have demonstrated that NFE2L2 knockdown in mice down-regulates the expression of several key mitochondrial-related genes.^35^ Further mechanistic studies have indicated that the promoter of NRF1 contains four ARE sequences, suggesting that NFE2L2 can enhance NRF1 transcription, thereby promoting MB.^36^ It has also been reported that PGC-1α, as a transcriptional coactivator, can amplify this transcriptional effect.^35^

Studies have shown that moderate overexpression of PGC-1α can enhance the number and function of mitochondria, particularly in subsarcolemmal mitochondria in muscle.^37^ Similarly, PGC-1α overexpression increases mitochondrial content and function in the liver, accompanied by enhanced fatty acid oxidation and reduced triglyceride storage and secretion.^38^ However, a recent study on the *Drosophila* model raised questions about the role of PGC-1α when Spargel (srl), the *Drosophila* homolog of the PGC-1 family, was examined using tko25t, a recessive mutant displaying global oxidative phosphorylation defects. The study found that srl overexpression did not effectively ameliorate the tko25t phenotype, suggesting that srl overexpression cannot improve mitochondrial function or increase mitochondrial quantity.^39^ This may indicate that the role of srl in the tko25t mutant is affected by translational or post-translational regulation or other growth inhibitory signals, which prevents the expected compensatory effects. Nonetheless, more research and additional models are required to further elucidate the function of PGC-1α.

Current research indicates a complex and finely tuned regulation of PGC-1α in the body. AMPK, an intracellular energy sensor, is activated under energy-deficient conditions. AMPK activates PGC-1α by directly phosphorylating it at threonine-177 and serine-538, thereby promoting MB and energy metabolism. This direct phosphorylation has been confirmed in skeletal muscle cells and mice.^40^ Additionally, AMPK can indirectly regulate PGC-1α function by increasing the expression and activity of SIRT1, a deacetylase that deacetylates and activates PGC-1α.^41^ The AMPK pathway can also increase PGC-1α expression and activity during exercise, thereby promoting MB, particularly in skeletal muscle. Notably, exercise-induced up-regulation of PGC-1α is dependent on AMPK activation.^42^

Moreover, Ca^2+^ plays a crucial role in regulating PGC-1α levels. Studies have shown that increasing Ca^2+^ concentration using Ca^2+^ release agents (*e.g.*, caffeine and W7) or Ca^2+^ ionophores (*e.g.*, ionomycin) can increase mitochondrial protein expression and function in mouse skeletal muscle. However, Calcium-calmodulin (CaM)-dependent protein kinase II (CaMKII) inhibitor KN93 can block Ca^2+^-induced MB. Further experiments have indicated that increased Ca^2+^ concentration significantly elevates the phosphorylation level of p38 mitogen-activated protein kinase (MAPK), suggesting p38 MAPK activation. Furthermore, Ca^2+^-induced p38 MAPK phosphorylation is significantly reduced when CaMKII activity is inhibited by KN93, indicating that p38 MAPK is downstream of CaMKII. Researchers (using the p38 MAPK inhibitor SB202190) have found that SB202190 significantly reduces Ca^2+^-induced PGC-1α expression and mitochondrial-related gene expression, confirming that Ca^2+^ may regulate MB via the Ca^2+^/CaMK/p38 MAPK/PGC-1α pathway.^43^ Furthermore, p38 MAPK can activate activating transcription factor 2 (ATF2), which binds to the CRE binding site on the PGC-1α promoter, promoting its transcription.^44^

**Figure 1:**
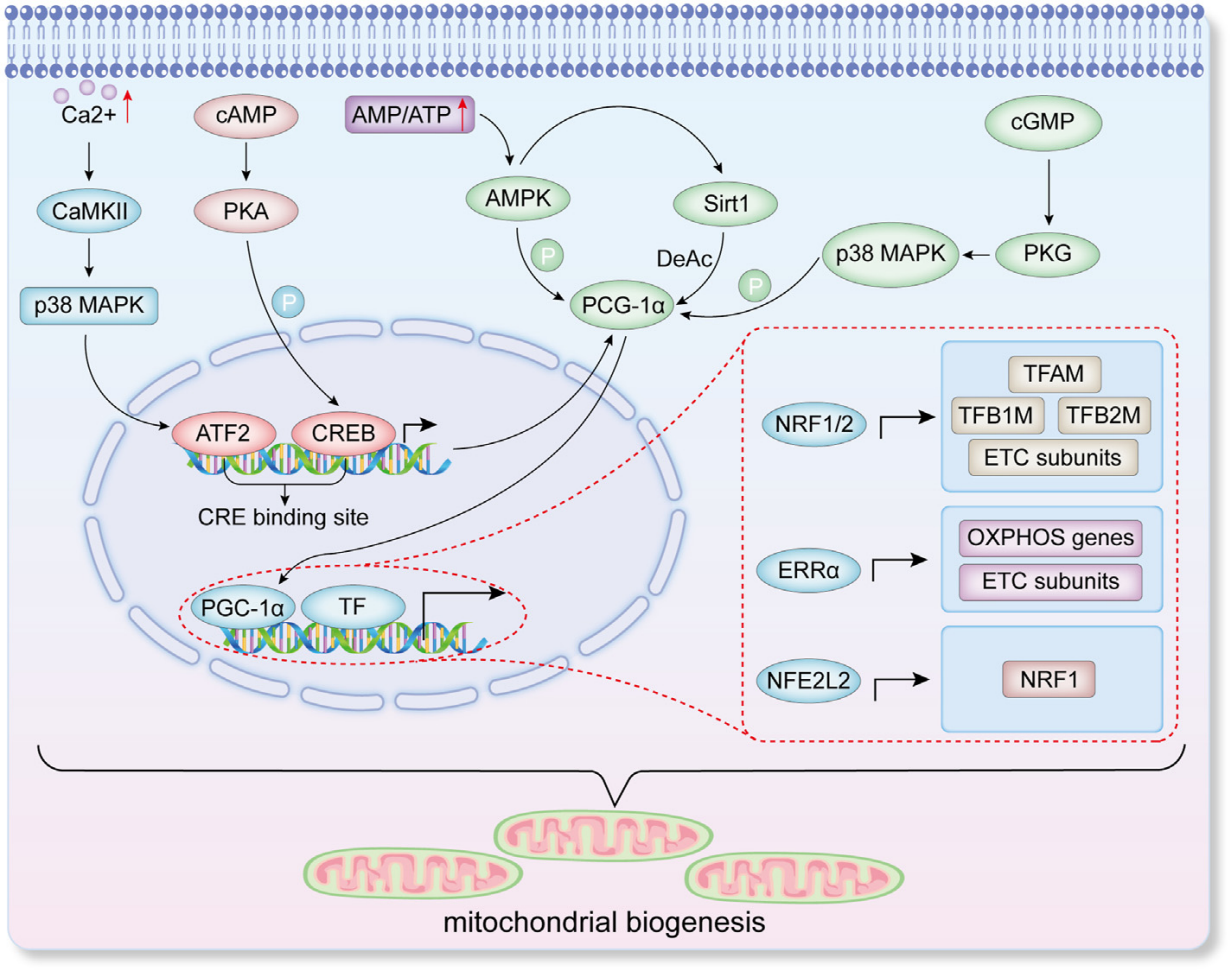
The pathways regulating mitochondrial biogenesis. CaMKII, calcium-calmodulin dependent protein kinase II; p38 MAPK, mitogen-activated protein kinase; cAMP, cyclic adenosine monophosphate; cGMP, cyclic guanosine monophosphate; PKA, protein kinase A; PKG, protein kinase G; AMPK, adenosine monophosphate-activated protein kinase; SIRT1, sirtuin 1; PGC-1α, peroxisome proliferator-activated receptor gamma coactivator 1-alpha; NRF1/2, nuclear respiratory factor 1/2; NFE2L2, nuclear factor erythroid 2-related factor 2; TF, transcription factor; TFB1M, transcription factor B1, mitochondrial; TFB2M, transcription factor B2, mitochondrial; TFAM, mitochondrial transcription factor A; ERRα, orphan nuclear hormone receptor; OXPHOS, oxidative phosphorylation; ETC, electron transport chain; DeAc, deacetylation; P, phosphorylation.

Additionally, cyclic adenosine monophosphate (cAMP) and cyclic guanosine monophosphate (cGMP), as intracellular second messengers, play key roles in regulating PGC-1α expression. Elevated cAMP levels activate protein kinase A (PKA), which then phosphorylates cAMP response element-binding protein (CREB) at Ser133. This phosphorylation site is essential for the transcriptional activity of CREB, allowing phosphorylated CREB to bind to CRE and initiating PGC-1α transcription, a crucial mechanism in muscle cells.^45^^,^^46^ cGMP influences CRE and AP-1 binding sites related to PGC-1α transcription by activating protein kinase G (PKG).^47^ Furthermore, cGMP can regulate the activity of phosphodiesterases (PDEs), impacting the cAMP signaling pathway. Specifically, cGMP can increase cAMP degradation and reduce cAMP degradation by activating PDE2 and inhibiting PDE3, respectively, thereby altering cAMP levels. This cross-regulation is significant for myocardial cell function.^48^ Understanding these complex signaling pathways and their interactions is crucial for studying intracellular energy metabolism and mitochondrial function regulation, providing potential targets and strategies for treating related diseases. MB regulatory mechanisms are shown in Figure 1.

## MB and AKI

MB is suppressed during the onset of AKI. The various pathological stimuli lead to the accumulation of damaged mitochondria while new mitochondria fail to synthesize, leading to an overall reduction in mitochondrial numbers. Decreased MB affects the function of renal tubular epithelial cells, which are rich in mitochondria, thus disrupting mitochondrial homeostasis. This imbalance directly or indirectly results in insufficient ATP production, increased reactive oxygen species (ROS), and excessive mitochondrial fragmentation.^49^^,^^50^ These adverse factors further deteriorate the function of renal tubular epithelial cells, exacerbating AKI.^51^

Numerous AKI models have demonstrated that MB is suppressed during AKI. For instance, key transcriptional regulators of MB, PGC-1α, and TFAM are significantly down-regulated in a mouse model of folic acid-induced AKI one day after folic acid injection and remain low for 14 days. Additionally, mtDNA copy numbers are decreased by about 50% from day 2–14 after folic acid injection.^52^ Furthermore, the activation of Toll-like receptor 4 (TLR4) and the MEK/ERK signaling pathway inhibits MB in endotoxin-induced AKI. However, lipopolysaccharide (LPS) can protect TLR4 knockout mice from kidney injury and MB suppression. Similarly, researchers found a 57% reduction in renal PGC-1α levels, accompanied by decreased mRNA levels and increased acetylation in a liver transplantation-induced AKI model. The TFAM protein and mRNA levels were decreased by 66% and 68%, respectively, further reducing mtDNA.^53^

Various stimuli trigger AKI, which may inhibit PGC-1α, TFAM, and other factors through different mechanisms, thereby suppressing MB. Nuclear factor kappa B (NF-κB) reduces PGC-1α expression and activity under inflammatory conditions through cytokines, such as TNF-α and IL-1β. The p65 subunit of NF-κB interacts with PGC-1α, reducing its activity and stabilizing this interaction, further inhibiting PGC-1α function.^54^ PGC-1β, a homolog of PGC-1α, shares similar tissue distribution and amino acid sequences and is equally critical for MB.^55^^,^^56^ C-MYC regulates PGC-1β expression. HIF-1α up-regulates MAX interactor 1 (MXI-1) expression under hypoxic conditions, promoting C-MYC degradation and inhibiting its transcriptional activity, thereby indirectly suppressing PGC-1β expression and MB.^57^ AMPK regulates mitochondrial gene expression and function by phosphorylating various epigenetic factors, such as DNA methyltransferase 1 (DNMT1) and histone acetyltransferase 1 (HAT1). Reduced AMPK activity suppresses PGC-1α and TFAM expression.^58^, ^59^, ^60^ Although AMPK activation is often triggered as a compensatory mechanism due to nutrient deficiency and energy shortage, the severe impact of AKI stimuli often inhibits AMPK, thus suppressing MB.^61^^,^^62^ TFAM is also inhibited during AKI. Besides, HK2 cells produce large amounts of mitochondrial ROS (mtROS) during ischemia-reperfusion (I/R)-AKI. mtROS inhibits TFAM transcription and promotes its degradation via Lon protease, reducing TFAM levels.^63^, ^64^, ^65^ These results indicate that targeting the activation of MB is a potential therapeutic strategy for AKI treatment. The following section summarizes and discusses the latest research targeting MB as a therapeutic approach for AKI, highlighting its therapeutic potential.

## Ischemia-reperfusion-induced AKI

I/R injury occurs when blood supply returns to tissue after ischemia or lack of oxygen. The interruption of blood flow during the ischemic phase leads to oxygen and nutrient deprivation in the kidney tissue. The reoxygenation of the tissue generates a large amount of ROS when blood flow is restored, which further exacerbates kidney damage.^66^^,^^67^ Targeting MB has emerged as an effective strategy to mitigate I/R-AKI. Numerous studies have shown that the expression of PGC-1α decreases during renal I/R, resulting in mitochondrial fragmentation and swelling. Forced overexpression of PGC-1α in renal tubular epithelial cells can increase mitochondrial abundance and respiratory capacity while reducing mitochondrial protein loss, thus alleviating AKI.^68^^,^^69^

Subsequent research has focused on upstream regulators of PGC-1α. Researchers have shown that the expression of PGC-1α and TFAM is suppressed in the renal cortex of I/R mouse models. Similarly, electron microscopy has revealed mitochondrial rupture, cristae disappearance, and increased autophagosome formation in hypoxia-reoxygenation models (using HK2 cells), indicating MB suppression. The transcription factor forkhead box O1 (FOXO1) is significantly up-regulated during I/R injury. FOXO1 overexpression through adenoviral transfection (Ad-FOXO1) can inhibit PGC-1α expression. Also, selective inhibitor AS1842856 can significantly inhibit FOXO1 expression induced by I/R and hypoxia-reoxygenation while increasing PGC-1α mRNA and protein levels. Co-immunoprecipitation experiments have demonstrated that FOXO1 inhibits PGC-1α transcription by competing with phosphorylated CREB for binding to CREB binding protein/EP300 (CBP/P300).^70^

General control of amino acid synthesis 5-like 1 (GCN5L1) is a mitochondria-specific acetyltransferase crucial for MB regulation.^71^ GCN5L1 knockdown improves the reduction of mtDNA copy numbers, decreases OXPHOS complex expression, and reduces ATP content in the renal cortex following I/R injury. Electron microscopy has revealed increased mitochondrial area and length, suggesting that GCN5L1-mediated TFAM acetylation inhibits TFAM binding to the mitochondrial outer membrane translocase TOM70, reducing TFAM entry into mitochondria, thereby inhibiting MB.^72^

**Figure 2:**
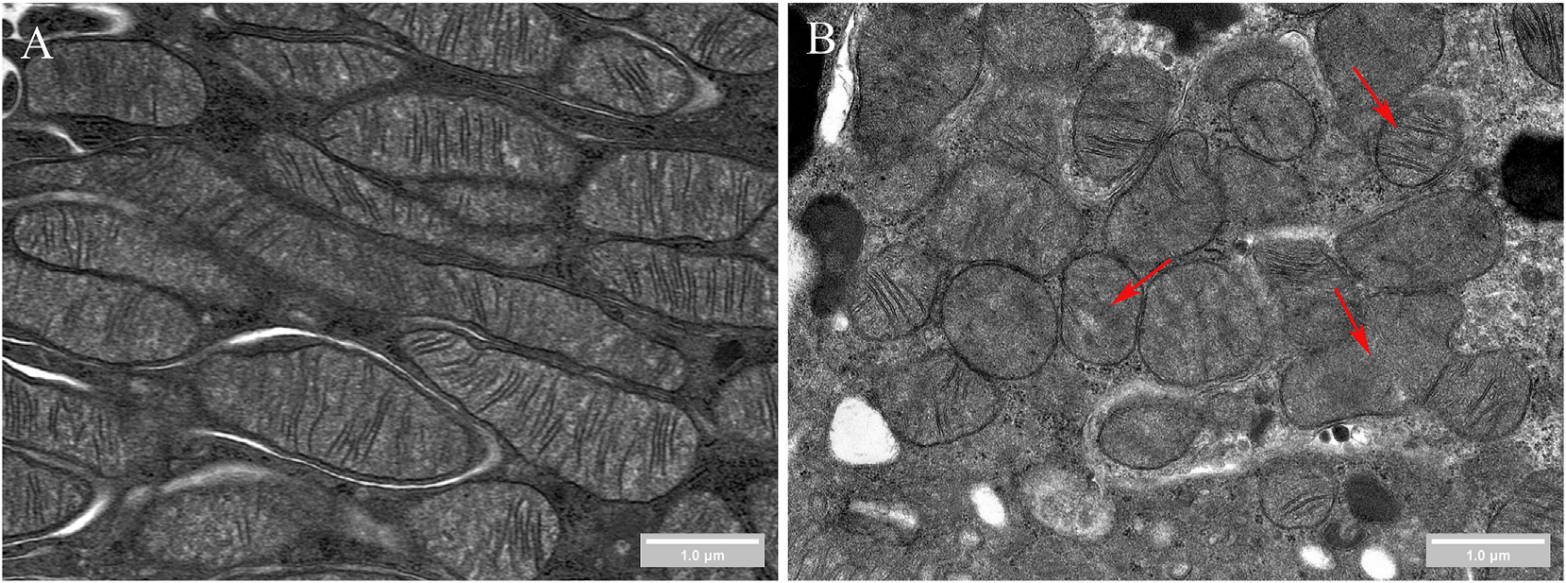
Mitochondrial damage in rat kidneys following ischemia-reperfusion. Panel A illustrates the mitochondrial morphology in proximal tubule cells of normal rat kidneys, characterized by elongated structures with well-defined and abundant cristae. In contrast, panel B depicts mitochondrial morphology in proximal tubule cells after 30 min of ischemia followed by 24 h of reperfusion. These mitochondria display swelling, fragmentation, and a notable reduction in density. The red arrows highlight disrupted cristae structures, marked by irregular arrangements and, in some instances, a complete loss of cristae. Such pathological alterations are consistently observed across the examined tissue sections. The red arrow at the bottom points to mitochondria exhibiting abnormal morphology, deviating from their typical linear or elliptical shapes, indicative of disrupted mitochondrial dynamics. These morphological and structural abnormalities contribute to mitochondrial dysfunction, exacerbating acute kidney injury. Importantly, strategies aimed at enhancing MB have been shown to mitigate these damages.

Besides the genetic approaches, researchers have focused on small molecule compounds to mitigate MB inhibition under I/R conditions. Lasmiditan, a selective 5-hydroxytryptamine 1F (5-HT1F) receptor agonist,^73^ induces MB by activating the 5-HT1F receptor. This activation increases PGC-1α levels in the renal cortex, restores mitochondrial protein levels, improves mitochondrial morphology, increases mitochondrial area and length, reduces mitochondrial damage, and restores mtDNA and ATP content.^74^ Another study found that lasmiditan (0.3 mg/kg) can increase mitochondrial numbers in the renal cortex by 1.4 times and reduce fibrosis and proximal tubular damage, further demonstrating that lasmiditan can enhance MB and alleviate AKI.^75^ The transmission electron microscopy (TEM) images of mitochondria during I/R-AKI are shown in Figure 2.

## Sepsis-induced AKI

Sepsis is a systemic inflammatory response caused by infection and can lead to multiple organ dysfunction, especially kidney dysfunction.^76^ The systemic inflammatory response often results in a cytokine storm and hyperactive immune system, causing AKI. MB exhibits time-dependent changes in sepsis-induced AKI (S-AKI). Early MB, as a compensatory mechanism, is activated to respond to damage. TEM shows minimal mitochondrial morphological changes 4 h post-cecal ligation and puncture, but significant changes, including swelling and cristae disruption, occur over time. Mitochondrial content and biogenesis are significantly decreased 24 h post-cecal ligation and puncture, evidenced by decreased mtDNA copy numbers and mRNA expression of PGC-1α and TFAM, indicating later suppression of MB.^77^

Sepsis often leads to severe inflammatory responses and cytokine storms, resulting in significant kidney damage and suppressed MB during S-AKI. Several studies have identified MB as a potential therapeutic target for preventing S-AKI.^78^, ^79^, ^80^ Moreover, the LPS-induced S-AKI suppressed PGC-1α expression in a mouse model, with the TEM examination indicating swollen mitochondria and cristae loss in LPS-induced S-AKI, suggesting MB suppression.^81^ Tubular β-catenin, an essential component of the cell adhesion complex,^82^ can partially restore these mitochondrial morphological changes. The tubule-specific β-catenin stabilized (TubCat) mice have reduced mitochondrial swelling, increased mitochondrial quantity and quality, and significantly higher expression of mitochondrial marker protein TIM23 and MB regulators PGC-1α and NRF1. Furthermore, blood urea nitrogen and creatinine levels are significantly reduced in TubCat mice, while tubular injury (NGAL-positive tubules) is decreased. In contrast, tubule-specific β-catenin deleted (TubCatKO) mice exhibit exacerbated damage, highlighting the critical role of tubular β-catenin in restoring mitochondrial quality by regulating MB through the FOXO3/PGC-1α signaling pathway.^78^

Malvidin, a natural plant compound belonging to anthocyanins, possesses antioxidant and anti-inflammatory properties.^83^ Malvidin can regulate MB in S-AKI. TEM has shown increased mitochondrial damage in S-AKI mice, including swelling, cristae disruption, and increased transparency. However, malvidin treatment can alleviate these damages, reduce ROS accumulation, and enhance mitochondrial membrane potential, thus maintaining mitochondrial homeostasis and mitigating renal injury. Further studies have indicated that malvidin can increase total and nuclear PGC-1α protein expression, directly activating AMPK and Sirt1, which activate PGC-1α through phosphorylation and deacetylation. This co-activation of downstream transcription factors NRF1/2 further activates TFAM.^79^

NFE2L2, a major regulator of cellular redox homeostasis, helps maintain mitochondrial morphology and function under stress conditions. *In vitro* models (LPS-treated NRK-52e cells) have shown that NFE2L2 overexpression can significantly increase PGC-1α and TFAM levels, indicating that NFE2L2 mitigates mitochondrial damage by promoting MB. The NFE2L2 agonist TBHQ can significantly restore the levels of PGC-1α, and TFAM decreased by cecal ligation and puncture. TEM has revealed extensive mitochondrial swelling, cristae disruption, and mitochondrial disintegration in LPS-treated NRK-52e cells and kidney tissues of cecal ligation and puncture modeled rats. However, NFE2L2 overexpression or activation can mitigate these damages, reducing mitochondrial deformation and rupture, indicating that NFE2L2 is a promising therapeutic target for S-AKI.^80^

## Cisplatin-induced AKI

Cisplatin is a widely used anti-cancer drug associated with nephrotoxicity, which leads to AKI.^84^ The cisplatin-induced nephrotoxicity is a complex, multifactorial pathological process involving oxidative stress, inflammation, apoptosis, mitochondrial dysfunction, and DNA damage.^85^ Nonetheless, hydration therapy, pharmacological protection, and dosage adjustments can mitigate cisplatin-induced nephrotoxicity, enhancing its clinical safety.

Studies using TEM to observe mitochondrial morphology and diameter in live cultured renal tubular epithelial cells under different treatments have shown that cisplatin treatment leads to abnormal mitochondrial changes, including mitochondrial swelling, vacuolation, and cristae fragmentation. The expression levels of PGC-1α and TFAM are significantly reduced in a model of cisplatin-induced AKI, suggesting that MB is suppressed during cisplatin treatment. However, liquiritigenin, a licorice extract, can significantly reduce renal damage in cisplatin-treated mice by lowering blood urea nitrogen and creatinine levels, improving renal function, reducing tubular injury, and reducing mitochondrial abnormalities. Liquiritigenin can also mitigate the suppression of cisplatin-induced MB by enhancing the expression of NFE2L2 and SIRT3, which promotes PGC-1α and TFAM expression, thereby improving MB and providing protective effects against cisplatin-induced AKI.^86^

Another study found that mitochondria in cisplatin-treated HKC-8 cells showed swelling, disrupted cristae alignment, and fragmentation. Furthermore, cisplatin treatment significantly suppressed PGC-1α and TFAM expression in renal tubular cells, indicating MB inhibition. However, penicilliumin B can alleviate the aforementioned damage and the suppression of PGC-1α and TFAM expression and maintain normal levels of translocase of outer mitochondrial membrane 20 (TOMM20), suggesting a protective effect on mitochondrial quality.^87^

## Folic acid-induced AKI

Folic acid-induced AKI is caused by high-dose folic acid intake or abnormalities in folic acid metabolism under certain disease conditions. This condition induces severe damage to renal tubules through various mechanisms, such as oxidative stress, inflammation, and cell death.^88^

In an earlier study, folic acid injection significantly decreased the mRNA expression of PGC-1α and TFAM in mice by about 80% after one day and remained low throughout the experiment. Also, mtDNA copy numbers decreased by about 50% from day 2 to day 14 post-injection. TEM showed significant mitochondrial swelling and loss of cristae structure in renal tubular epithelial cells one day after folic acid treatment, persisting until the end of the 14-day observation period, indicating MB suppression.^52^

MB suppression may exacerbate folic acid-induced AKI damage. Researchers found that PGC-1α deficient mice (Ppargc1a^−/−^ mice) exhibit more severe loss of mitochondrial quality and function during folic acid-induced AKI, as evidenced by the down-regulation of mitochondrial-related genes (Tfam, Ndufs1, and Sdha), reduced TOMM22 staining, and decreased mtDNA, which are correlated with higher levels of kidney injury and inflammation. However, the study did not construct PGC-1α-overexpressing mice for a positive confirmation, thus lacking some degree of persuasiveness.^89^

In another study, PGC-1α mRNA and protein levels and the expression of mitochondrial-related genes (Ndufs1, Sdha, and Tfam) were reduced in mice with folic acid-induced AKI, indicating MB suppression. This suppression exacerbated kidney damage. Besides, the injection of tumor necrosis factor-like weak inducer of apoptosis (TWEAK), a cytokine of the tumor necrosis factor superfamily, aggravated this damage. Systemic injection of TWEAK reduced PGC-1α mRNA and protein levels in the kidney of healthy mice. Conversely, a neutralizing antibody against TWEAK can prevent the decline in PGC-1α and its target gene expression during AKI and mitigate folic acid-induced AKI damage.^90^ Further studies have shown that TWEAK down-regulates PGC-1α expression through NF-κB activation and histone deacetylation mechanisms, affecting MB and leading to mitochondrial dysfunction and further kidney damage.^91^

## AKI induced by other factors

In a study investigating mitochondrial homeostasis disruption in rat AKI following liver transplantation, researchers found that the levels of primary MB regulator, PGC-1α, decreased by 57%, with its mRNA levels reduced by 63% and its activity inhibited. Furthermore, oxidative phosphorylation (OXPHOS) proteins ATP synthase-β and NADH dehydrogenase-3 were reduced by 44% and 81% in the rat kidneys, respectively, along with a significant reduction in their associated mRNAs, indicating impaired mitochondrial function. Also, blood urea nitrogen and creatinine levels doubled post-transplantation, indicating kidney damage. Pathological changes ranged from mild to moderate, including brush border loss, vacuolization of cortical tubular cells, and necrosis in some proximal tubular cells. These findings suggest that MB suppression and mitochondrial dysfunction may lead to AKI post-liver transplantation. However, further research is needed to assess whether enhanced MB can mitigate AKI.^53^

In another experiment, maleic acid treatment significantly reduced levels of NRF1, NRF2, PGC-1α, and TFAM in both mice and NRK-52E cells in AKI models. TEM observations showed extensive mitochondrial fragmentation and cristae loss. However, sulforaphane treatment alleviated these damages and mitigated the decrease in fatty acid-related oxygen consumption rate, OXPHOS capacity, mitochondrial membrane potential, and complex I activity, suggesting the protective ability of sulforaphane against maleic acid-induced AKI.^92^

Gentamicin often induces AKI due to the presence of free radicals.^93^ Besides, blood urea nitrogen and creatinine levels are significantly increased in rats with gentamicin-induced AKI, with TEM showing marked abnormalities in mitochondrial morphology and quantity. Furthermore, PGC-1α, p-PKA, and p-CREB protein expressions are significantly decreased in rats with gentamicin-induced AKI, indicating MB suppression. Further investigation revealed that gentamicin can significantly increase Notch1 and Hes-1, pathways that further inhibit PGC-1α expression and MB. However, pretreatment with liraglutide, a human glucagon-like peptide-1 analogue,^94^ can mitigate these damages. Mechanistic studies have suggested that liraglutide promotes MB by enhancing PKA/CREB and inhibiting Notch/Hes-1 signaling pathways.^95^ These findings are summarized in Table 1.

**Table 1:** Targeting mitochondrial biogenesis alleviates acute kidney injury.

Disease model	Treatment method	Subjects	Regulatory mechanism	Experimental results	References
I/R-AKI	Overexpression of PGC-1α	New Zealand White rabbits, RPTCs cells	Overexpression of PGC-1α caused a 52% increase in mitochondrial number, a 27% enhancement in respiratory capacity, and a 30% elevation in mitochondrial protein markers.	The increase in PGC-1α expression led to a rise in basal oxygen consumption on the second day and an increase in uncoupled oxygen consumption on the third day, both of which returned to control levels by the fourth day.	Rasbach and Schnellmann^68^
I/R-AKI	Generation of renal tubular PGC-1α transgenic mice	C57BL/6J mice	PGC-1α regulates mitochondrial biogenesis (MB) and the biosynthesis of nicotinamide adenine dinucleotide (NAD).	iNephPGC1α promoted MB increase, enhanced local NAD levels, and reduced fat accumulation, thereby improving kidney function.	Tran et al^69^
I/R-AKI	FOXO1 selective inhibitor AS1842856	C57BL/6 mice, HK2	FOXO1 inhibits MB by suppressing the transcription of PGC-1α via competing with cAMP response element-binding protein (CREB) for binding to CBP/P300.	The FOXO1 selective inhibitor AS1842856 increased the expression of mitochondrial biogenesis-related proteins, such as PGC-1α and TFAM, reduced serum urea nitrogen and creatinine levels, and improved the survival rate of mice.	Wang et al^70^
I/R-AKI	GCN5L1 knockdown	C57BL/6J mice, TECs	GCN5L1 can acetylate TFAM at the K76 site, inhibiting its binding to TOM70, reducing TFAM entry into mitochondria, and thereby affecting MB.	In the context of GCN5L1 knockdown, AKI-induced mitochondrial damage was alleviated, as evidenced by decreased serum creatinine and urea nitrogen levels and increased mitochondrial number and function.	Lv et al^72^
I/R-AKI	Lasmiditan	C57BL/6NCrl mice	Lasmiditan improves kidney function by promoting MB through activation of the 5-hydroxytryptamine 1F receptor (5-HT1F).	Lasmiditan treatment reduced mitochondrial damage, increased mitochondrial area and size, and restored normal mitochondrial morphology. Additionally, lasmiditan treatment increased ATP content and mitochondrial DNA quantity in the renal cortex.	Hurtado et al^74^
I/R-AKI	Lasmiditan	C57BL/6NCrl mice, TECs	Lasmiditan promotes mitochondrial biogenesis through the activation of 5-HT1F.	In the lasmiditan treatment group, the number of mitochondria in the renal cortex of mice increased by 1.4 times, and creatinine levels decreased.	Hurtado et al^75^
S-AKI	Overexpression of β-catenin	C57BL/6J mice, HK2	The stabilization of β-catenin significantly increased the expression of PGC-1α and NRF1, restoring mitochondrial quality.	PGC-1α and NRF1 expression were increased in TubCat mice, along with elevated TIM23 levels, increased mitochondrial number, more intact morphology, restored ATP levels, and mitochondrial DNA replication.	Li et al^78^
S-AKI	Malvidin	C57BL/6 mice, HK2	Malvidin regulates MB by promoting the nuclear translocation of PGC-1α and its interaction with Nrf2.	Malvidin significantly restored LPS-induced mitochondrial membrane potential decline, reduced ROS production, increased mitochondrial DNA copy number, and improved mitochondrial quality and function, thereby alleviating kidney injury.	Fan et al^79^
S-AKI	NFE2L2 activation	Sprague–Dawley rats, NRK-52e	NFE2L2 promotes the PGC-1α and TFAM expression, enhancing MB.	NFE2L2 activation increased the expression of proteins related to mitophagy and mitochondrial biogenesis, reduced mitochondrial damage, and restored normal mitochondrial structure.	Chen et al^80^
Cis-AKI	Liquiritigenin	BALB/c mice, HK2	Liquiritigenin promotes the expression of PGC-1α and TFAM by enhancing the expression of NFE2L2 and SIRT3.	Liquiritigenin improved mitochondrial function and inhibited apoptosis through the NFE2L2/SIRT3 pathway, thereby ameliorating kidney injury.	Zhou et al^86^
Cis-AKI	Penicilliumin B	C57BL/6 mice, HKC-8	Penicilliumin B promotes autophagy and MB by activating the adenosine monophosphate-activated protein kinase (AMPK) pathway.	Penicilliumin B improved mitochondrial morphology in cisplatin-treated HKC-8 cells, significantly inhibited the cisplatin-induced up-regulation of the mitochondrial fission protein Drp1, and restored the expression of TFAM.	Shen et al^87^
FA-AKI	Ppargc1a^−/−^ mice	C57BL/6 mice, MCT	PGC-1α promotes MB.	After PGC-1α knockout, Tfam, Ndufs1, and Sdha were found to be down-regulated, TOMM22 staining was reduced, and mitochondrial DNA decreased, along with higher levels of kidney injury and inflammation.	Fontecha-Barriuso et al^89^
FA-AKI	TWEAK	C57BL/6 mice, MCT	TWEAK down-regulates PGC-1α expression via NF-κB activation and histone deacetylation mechanisms.	TWEAK down-regulated PGC-1α, leading to reduced expression of PGC-1α target genes such as Ndufs1, Sdha, and Tfam, thereby impairing mitochondrial function.	Ruiz-Andres et al^91^
MA-AKI	Sulforaphane	Wistar rats, NRK-52E	Sulforaphane significantly increases NRF1, NRF2, PGC1α, and TFAM expression.	Sulforaphane significantly improved maleic acid-induced mitochondrial membrane potential (Ψmt) decline and restored mitochondrial oxidative phosphorylation capacity as well as the enzymatic activity of mitochondrial complexes I and IV.	Briones-Herrera et al^92^
GM-AKI	Liraglutide	Sprague–Dawley rats	Liraglutide promotes MB by enhancing the expression of PGC-1α, PKA, and CREB and inhibiting the expression of Notch1 and Hes-1.	Transmission electron microscopy revealed improved renal mitochondrial morphology in the liraglutide-treated rats, with an increased number and structure close to normal.	Elkhoely^95^

Note: I/R-AKI, ischemia-reperfusion acute kidney injury; S-AKI, sepsis-associated acute kidney injury; Cis-AKI, cisplatin-induced acute kidney injury; FA-AKI, folic acid-induced acute kidney injury; MA-AKI, maleic acid-induced acute kidney injury; GM-AKI, gentamicin-induced acute kidney injury; RPTCs, renal proximal tubular cells; iNephPGC1α, inducible nephron progenitor cell PGC1α transgenic mice; TECs, tubular epithelial cells; TubCat, tubular cell-specific β-catenin stabilization; Drp1, dynamin-related protein 1; MCT, murine cortical tubular cells; GCN5L1, general control of amino acid synthesis 5-lik TWEAK, tumor necrosis factor-like weak inducer of apoptosis.

## The compounds targeting MB

Several interventions targeting AKI involve genetic editing to alter levels of MB-related proteins. However, the clinical application of genetic editing poses certain difficulties and challenges. This section summarizes promising small-molecule compounds that directly target the MB protein. This approach holds potential for future clinical applications due to the critical role MB plays in various cellular processes.

As a coactivator, PGC-1α lacks a nucleic acid binding domain and possesses ligand binding sites,^96^^,^^97^ making it difficult to identify compounds that directly target and activate PGC-1α. Traditional high-throughput screening methods are usually based on known ligand binding sites or enzymatic activity, which do not apply to PGC-1α. Due to the complexities associated with directly targeting PGC-1α, the search for small molecule modulators often prioritizes its upstream regulatory network. This network includes potential transcription factors and deacetylation-inducing factors, which play a crucial role in activating PGC-1α.

Pyrroloquinoline quinone is a small molecule compound that is involved in mitochondrial function and biogenesis. In Hepa1-6 mouse liver cell lines exposed to 10–30 μM pyrroloquinoline quinone, the activity of citrate synthase and cytochrome c oxidase was increased, accompanied by enhanced MitoTracker staining, mtDNA content, and cellular oxygen consumption, indicating increased MB. Further analysis using western blotting revealed an increase in both the expression and phosphorylation of PGC-1α and CREB proteins. This finding was corroborated by reporter assays, which showed heightened activity of the transcription factors NRF-1, NRF-2, and CREB. These results collectively support the notion that MB enhances the activity of these important cellular regulators. Using a reporter plasmid containing the PGC-1α promoter region, pyrroloquinoline quinone exposure induced CREB phosphorylation at serine 133, activating the PGC-1α promoter and up-regulating the PGC-1α mRNA and protein expression level.^98^

ZLN005, a novel small molecule with a benzimidazole core structure,^99^ can up-regulate the PGC-1α expression by activating the AMPK, markedly enhancing MB in muscle and liver cells. In diabetic db/db mice, ZLN005 significantly promoted the PGC-1α transcription in skeletal muscle and its downstream genes while down-regulating the PGC-1α and gluconeogenic gene expression in the liver, improving glucose tolerance, insulin sensitivity, and lipid metabolism.^100^

Peroxisome proliferator-activated receptor gamma (PPARγ), due to its widespread distribution in various brain regions and anti-inflammatory properties, may be a potential target for conferring neuroprotection in neurodegenerative diseases such as Alzheimer's and Parkinson's diseases.^101^^,^^102^ PGC-1α, as a coactivator of PPAR-γ,^103^ can participate in neuroprotective mechanisms. In a previous study, bioisosteric principles and computer-aided design were employed to synthesize two novel thiazolidinediones (G1 and G2). Pre-treatment with G1/2 reduced LPS-induced damage in SH-SY5Y neuroblastoma cells. Mechanistic studies revealed that G1 and G2 activate the PPAR-γ signaling pathway by binding to the PPAR-γ receptor, forming a heterodimer with the retinoid X receptor (RXR), and recruiting PGC-1α to the nucleus. This regulation of multiple target genes provides neuroprotective effects.^104^

Resveratrol increases PGC-1α activity and MB in mouse muscle fibers primarily by decreasing PGC-1α acetylation and enhancing PGC-1α activity.^105^ Quercetin improves mitochondrial function and suppresses oxidative stress in diabetic neuropathy models by activating the AMPK/PGC-1α signaling pathway.^106^

A recent study provided new strategies for developing and identifying compounds targeting PGC-1α. PGC-1α1, a specific variant of the PGC-1α family, mainly modulates MB and oxidative metabolism, promoting energy expenditure and metabolic adaptation.^107^ Controlled by multiple E3 ubiquitin ligases, PGC-1α1 is continuously tagged for degradation through ubiquitination, limiting its accumulation in cells. Furthermore, PGC-1α1 lacks a stable three-dimensional structure, making it more prone to recognition and degradation by the 20S proteasome, which affects its cellular stability and function.^108^^,^^109^ Therefore, increasing protein stability and extending the half-life of PGC-1α1 to enhance its biological functions has become a new strategy for identifying compounds targeting PGC-1α1.^110^ A cell-based high-throughput screening system was developed using HEK 293 cells expressing an EGFP-PGC-1α1 fusion protein. This system identifies potential PGC-1α1 stabilizers by measuring the intensity and subcellular distribution of the EGFP-PGC-1α1 signals. Screening 7040 small molecule compounds identified four compounds (AM31, AM73, AM79, and AM80) that stabilize PGC-1α1 protein levels, prolonging its active duration and enhancing its biological effects.^111^ However, we did not explore how these four compounds stabilized PGC-1α1, and this needs to be further investigated.

Lon protease is an ATP-dependent protease that regulates protein quality control in mitochondria, selectively degrading misfolded proteins and specific short-lived regulatory proteins.^111^ TFAM, a crucial regulator of mtDNA replication and transcription, is rapidly degraded by Lon protease when it is not bound to mtDNA.^64^ Tetramethylpyrazine, a major component of *Ligusticum wallichii*, reportedly binds directly to TFAM. This binding may alter TFAM's conformation, preventing its recognition and degradation by Lon protease. Consequently, TFAM accumulates in cells, thereby promoting MB.^112^

NRF1, which is a membrane-bound protein in the endoplasmic reticulum, is continuously transported and degraded by the proteasome via ATPase p97.^113^ A decrease in proteasome activity triggers the NRF1 to undergo proteolytic cleavage by DNA damage inducible 1 homolog 2 (DDI2), allowing its nuclear translocation to promote MB transcription. High-throughput screening identified 45 compounds that could increase PSMA4-ARE-LUC signals more than threefold in a dose-dependent manner. Among them, sCGA884 and sCIN027 did not inhibit the ubiquitin-proteasome system but significantly activated NRF1 transcription responses, suggesting that they are potential new targets involved in NRF1 regulatory mechanisms. They can be used to develop NRF1-targeted drugs.^114^

Moreover, dimethyl fumarate modifies cysteine residues of Kelch-like ECH-associated protein 1 (Keap1), particularly at C151, C273, and C288 positions, mediated by its metabolite monomethyl fumarate. This modification alters the Keap1's conformation, preventing strong binding to NFE2L2, which promotes NFE2L2 dissociation and nuclear translocation to enhance MB.^115^

**Figure 3:**
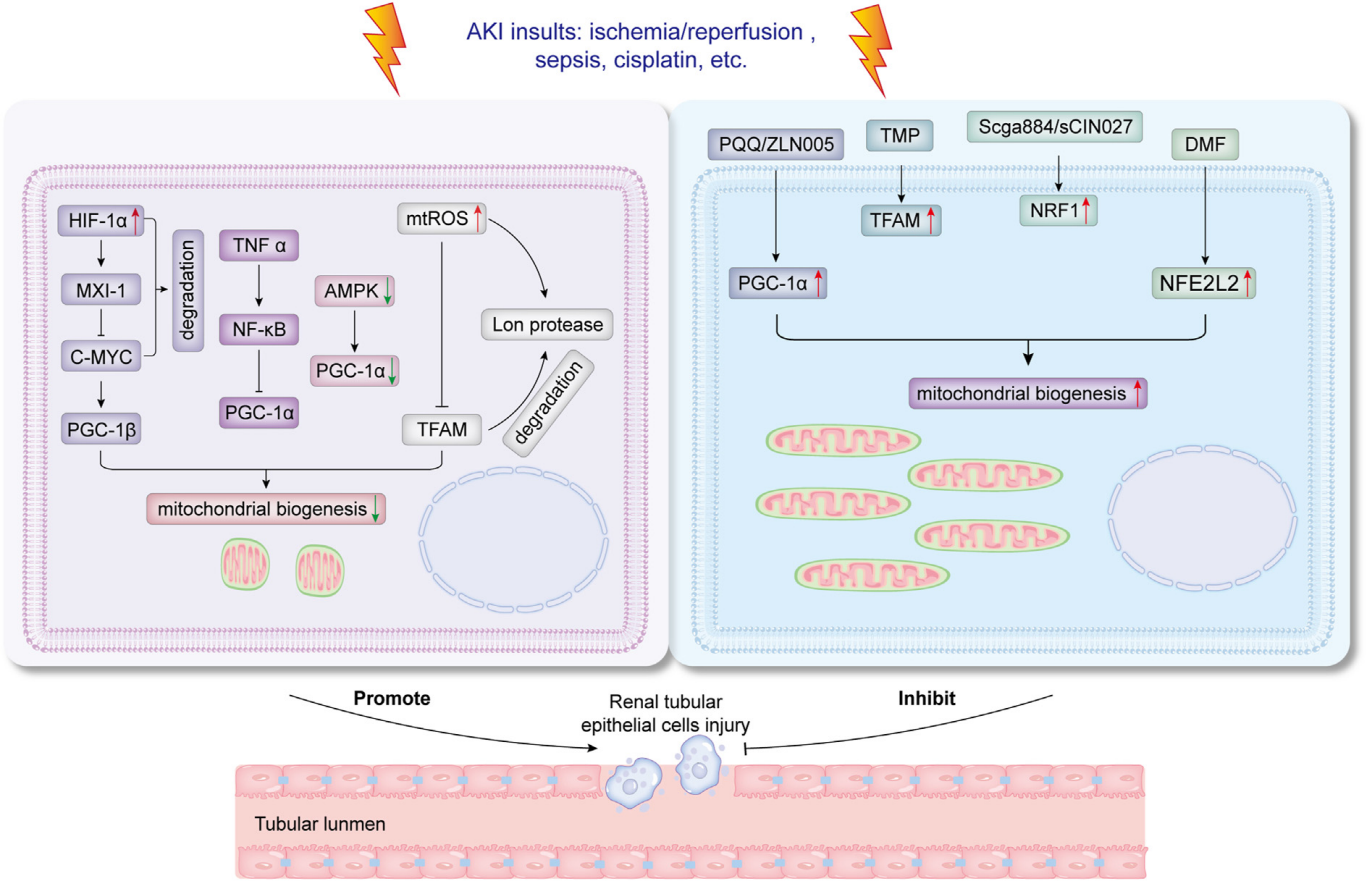
Modulation of mitochondrial biogenesis to alleviate acute kidney injury. This figure illustrates the mechanisms by which mitochondrial biogenesis is inhibited during acute kidney injury. It also demonstrates how targeting mitochondrial biogenesis with compounds such as pyrroloquinoline quinone (PQQ) and tetramethylpyrazine (TMP) can enhance mitochondrial biogenesis and thereby alleviate acute kidney injury-induced damage. PGC-1α/β, peroxisome proliferator-activated receptor gamma coactivator 1-alpha/beta; mtROS, mitochondrial reactive oxygen species; AMPK, adenosine monophosphate-activated protein kinase; NRF1, nuclear respiratory factor 1; NFE2L2, nuclear factor erythroid 2-related factor 2; TFAM, mitochondrial transcription factor A; DMF, dimethyl fumarate.

In summary, developing and identifying compounds targeting MB present multiple challenges. MB is regulated by a variety of factors, including PGC-1α, TFAM, NRF1, and NFE2L2, creating a complex network that complicates the development process. Secondly, it is difficult to target MB compounds without affecting other biological processes. While high-throughput screening tools are widely employed, their effectiveness and reliability are limited. Moreover, the lack of reliable biomarkers for assessing MB activity limits drug development. Challenges such as drug delivery, individual variability, and long-term safety must be addressed to facilitate clinical translation. To enhance readability, we summarized this content in Table 2. Moreover, we described the inhibition of MB in AKI and the rescue of MB via various compounds to alleviate AKI in Figure 3.

**Table 2:** The compounds targeting mitochondrial biogenesis.

Compounds	Subjects	Targets	Mechanisms	References
Pyrroloquinoline quinone (PQQ)	Hepa1-6 cells	PGC-1α	Exposure to PQQ induces CREB phosphorylation at serine 133, activating the PGC-1α promoter.	Chowanadisai et al^98^
ZLN005	db/db mice	PGC-1α	Up-regulates PGC-1α expression by activating AMPK.	Zhang et al^100^
G1/G2	SH-SY5Y cells	PGC-1α	G1 and G2 activate the PPAR-γ signaling pathway by binding to the PPAR-γ receptor. The activation of the PPAR-γ receptor promotes the formation of a heterodimer with the retinoid X receptor (RXR) and recruits PGC-1α into the nucleus.	Justin et al^104^
Resveratrol	C57Bl/6J mice/KKAy mice	PGC-1α	Increases SIRT1 activity and deacetylates PGC-1α.	Lagouge et al^105^
Quercetin	Sprague–Dawley rats	PGC-1α	Increases the AMP/ATP ratio, activates AMPK, and promotes its phosphorylation. Following activation, AMPK phosphorylates PGC-1α.	Zhang et al^106^
AM31, AM73, AM79, AM80	HEK 293 cells	PGC-1α1	Stabilizes PGC-1α1 protein, increases its expression in the cell, thereby prolonging its active duration and enhancing its biological effects.	Pettersson-Klein et al^110^
Tetramethylpyrazine	HeLa, EC-1, HCT116 cells	TFAM	Directly binds to TFAM, making it less susceptible to recognition and degradation by Lon protease.	Lan et al^112^
sCGA88, sCIN027	HepG2, HEK293T cells	NRF1	Activates NRF1 transcription.	Iaconelli et al^114^
Dimethyl fumarate	C57Bl/6J mice	NFE2L2	Forms covalent modifications with Cys151, Cys273, and Cys288 residues of Keap1, and promotes the dissociation of NFE2L2 and its translocation into the nucleus.	Hayashi et al^115^

## Clinical translation of mitochondrial-targeted therapies for AKI

### Clinical research on mitochondria-targeted therapies for alleviating AKI

AKI constitutes a major clinical challenge in nephrology globally. Its etiology is diverse, with common triggers encompassing surgical stress, hypovolemia, nephrotoxicity, and systemic inflammatory responses. Current treatment strategies primarily focus on symptomatic support, yet these approaches often fail to halt the progression of pathological processes and prevent further structural and functional damage to the renal tissue. Furthermore, the high heterogeneity among AKI patients, in terms of age and comorbidities, creates high variability in disease progression, and no effective targeted therapeutic options have been developed.^116^ As a high-energy-demand organ, the kidney relies heavily on mitochondrial function, particularly in tubular epithelial cells, for normal functioning.^117^ Mitochondrial dysfunction contributes to the development and progression of AKI, suggesting that therapies that improve mitochondrial stability may be effective in preventing AKI deterioration.^51^

Early identification and reversal of mitochondrial damage, such as via the suppression of excessive mtROS production, stabilization of mitochondrial membrane potential, enhancement of mitochondrial quality control and MB, or specific molecular interventions targeting mitochondrial respiratory chain complexes, may disrupt the disease process at the early stage. Consequently, mitochondria-targeted therapeutic strategies offer a novel, highly specific, and potentially restorative approach to managing AKI.

Evidence from previous studies demonstrates that mitochondria-targeted therapies can potentially alleviate AKI. For instance, elamipretide (also known as bendavia), a mitochondria-targeted drug, selectively binds to cardiolipin in the inner mitochondrial membrane, inhibiting its peroxidation, preserving mitochondrial function, and promoting MB.^118^ In a phase IIa randomized, double-blind, placebo-controlled trial involving 14 patients with severe atherosclerotic renal artery stenosis, elamipretide was administered following percutaneous transluminal renal angioplasty, a procedure that often induces I/R injury and AKI. It was observed that elamipretide increased the estimated glomerular filtration rate, reduced serum creatinine levels, and enhanced renal blood flow while mitigating postoperative tissue hypoxia and systolic blood pressure to improve renal function compared with the control group.^119^

In a multi-center, randomized, double-blind, parallel-group phase IIa trial targeting post-cardiac surgery AKI, patients received five intravenous doses of recombinant alpha-1-microglobulin (RMC-035) within 48 h before and after surgery. RMC-035 targets mitochondria, particularly complex I of the respiratory chain, where it scavenges free radicals generated during mitochondrial respiration to preserve mitochondrial structure and function.^120^ Although RMC-035 did not significantly reduce the short-term incidence of postoperative AKI, it markedly improved renal function (estimated glomerular filtration rate increased by 4.3 mL/min/1.73 m^2^ from baseline) and reduced major adverse kidney events at 90 days, demonstrating potential long-term protective effects.^121^

Coenzyme Q10, a core component of the mitochondrial inner membrane electron transport chain, ensures efficient ATP production during oxidative phosphorylation by maintaining the effectiveness of electron transfer.^122^ In a randomized, double-blind, controlled clinical trial, patients were administered 200 mg coenzyme Q10 daily for one week before and after extracorporeal shock wave lithotripsy (ESWL). This yielded a significant improvement in estimated glomerular filtration rate and a reduction in urinary albumin-to-creatinine ratios and the serum β2-microglobulin levels (*p* < 0.05), with these benefits persisting after extracorporeal shock wave lithotripsy.^123^ These findings underscore the high clinical value of mitochondria-targeted therapies.

### The strategies to facilitate translational medicine

Although mitochondria-targeted therapies have demonstrated significant potential in pre-clinical studies, their adoption in clinical settings faces numerous challenges, which calls for further studies to accelerate its wide use. The available studies on MB and mitochondrial targeting were mainly conducted on animal and cell models, and thus, findings from such studies cannot be extrapolated to clinical settings. To bridge this gap, researchers should aim to develop more effective translational strategies.

One promising approach is the adoption of advanced model systems, such as organoids and humanized mouse models, which more accurately reflect the pathological features and therapeutic responses of human AKI. These models can enhance the clinical relevance of research findings. Additionally, incorporating biomarkers associated with human AKI (*e.g.*, NGAL and KIM-1) into study designs, coupled with patient data, machine learning, and data modeling, may help to accurately predict the potential efficacy of experimental interventions in patients, thereby improving clinical translatability.

Moreover, developing precise drug delivery systems is critical for the success of mitochondria-targeted therapies. Notably, most of the proposed delivery systems have several limitations, such as rapid renal clearance and metabolism, insufficient targeting specificity, minimal mitochondrial localization, tubular obstruction, and inflammation-induced fibrosis. Moreover, some delivery carriers may increase nephrotoxicity, which undermines the therapeutic efficacy. To address these challenges, emerging nanotechnology offers promising solutions to these issues. For instance, nanocarrier systems targeting CD44 receptors were reported to improve drug accumulation in damaged tubular cells,^124^ while triphenylphosphonium-based nanostructures responsive to mitochondrial membrane potential enhanced drug release in specific microenvironments, improving mitochondrial specificity.^125^

The potential side effects associated with mitochondria-targeted drugs also need to be addressed. For example, excessive clearance of mtROS could disrupt normal cellular signaling and impair renal repair processes.^126^ In the future, researchers should investigate optimal dosages and determine the long-term safety through clinical trials. Furthermore, the accumulation of mitochondria-targeted drugs in non-target organs, such as the heart or brain, may induce adverse effects. Therefore, enhancing drug selectivity or adopting targeted delivery systems can reduce this risk.^127^

Given the considerable heterogeneity in AKI etiology and subtypes, individualized treatment strategies tailored to specific subtypes need to be formulated. For instance, mitochondrial sublethal damage in S-AKI was found to exhibit a better response to antioxidant therapies, while I/R-AKI benefited more from interventions that enhance MB.^128^ Additionally, multi-omics data (*e.g.*, genomics, proteomics, and metabolomics) should be used to identify specific biomarkers and patient subgroups that are most suitable for mitochondria-targeted therapies. Comorbidities, such as diabetes and hypertension, often present in AKI patients, may achieve higher efficacy for these therapies. For example, the mitochondrial dysfunction inherent in diabetic patients may alter drug responses in such patients.^129^

Finally, the short natural course of AKI presents challenges in designing clinical trials with sufficient statistical power, including patient recruitment, endpoint selection, and efficacy evaluation criteria. Thus, multidisciplinary collaboration, early adaptive trial designs, and integration of real-world data are advocated to accelerate translational research in this field.

In summary, while mitochondria-targeted therapies have shown great promise in the treatment of AKI, bridging the gap from animal models to clinical applications requires sustained efforts focusing on model optimization, design of delivery systems, formulation of individualized strategies, clinical trials, and other critical areas. Addressing these challenges will pave the way for the development of more effective treatments for AKI.

## Conclusion and future outlook

MB participates in the maintenance of cellular energy metabolism, thereby modulating the response to environmental stress and enhancing cell survival. It presents broad application prospects in various scenarios, including the management of endocrine diseases, neurodegenerative diseases, cardiovascular diseases, and cancer.^130^, ^131^, ^132^, ^133^ Given the kidney's high-energy demand and the renal tubular epithelial cells' high metabolic requirements, targeting MB in AKI holds substantial promise and relevance. Recent studies underscore the importance of MB as a significant approach for mitigating AKI damage, presenting a novel therapeutic avenue.

Despite significant advancements in targeting MB for AKI treatment, several challenges remain. Firstly, the efficacy of these approaches needs further validation across various AKI models and clinical settings. Secondly, drugs targeting mitochondria may have unintended side effects, thus altering organ function. Therefore, a deep understanding of the regulatory mechanisms of MB and the optimization of therapeutic strategies is crucial.

In conclusion, targeting MB may generate new ideas and strategies for treating AKI. In the future, researchers should aim to optimize drug design, elucidate mechanisms of action, and validate clinical trials to achieve the widespread clinical application of this strategy. In this way, they will improve the prognosis and quality of life for AKI patients.

## CRediT authorship contribution statement

**Yajie Hao:** Writing – original draft. **Fahui Chen:** Formal analysis. **Xiya Ren:** Data curation. **Xiu Huang:** Investigation. **Xiaoshuang Zhou:** Conceptualization.

## Conflict of interests

The authors declare that they have no known competing financial interests or personal relationships that could have appeared to influence the work reported in this paper.
